# Patients' and radiographers' experiences of dose reducing abdominal compression in radiographic examinations—A qualitative study

**DOI:** 10.1002/nop2.439

**Published:** 2020-01-07

**Authors:** Oili Piippo‐Huotari, Eva Funk, Håkan Geijer, Agneta Anderzén‐Carlsson

**Affiliations:** ^1^ Department of Radiology Faculty of Medicine and Health Örebro University Örebro Sweden; ^2^ School of Health Sciences Faculty of Medicine and Health Örebro University Örebro Sweden; ^3^ University Health Care Research Center Faculty of Medicine and Health Örebro University Örebro Sweden

**Keywords:** compression device, patient experience, radiographer, radiological examination

## Abstract

**Aim:**

To describe patients' and radiographers' experiences of abdominal compression using conventional and patient‐controlled compression methods.

**Design:**

Qualitative descriptive design.

**Methods:**

Forty‐five patients who had used both a conventional and a patient‐controlled compression device answered questionnaires. Five radiographers were interviewed. The data‐collection took place between September 2015 and February 2017. Data were analysed by qualitative content analysis.

**Results:**

Patient‐controlled compression was preferred by slightly more patients because of fear of pain due to excessively hard pressure, maintaining control over the pressure and shorter duration. It was more comfortable, and patients felt they could participate in the examinations. Conventional compression was preferred by some because of more stable pressure and uncertainty of own capacity to provide the optimal compression. Discomfort was more often mentioned concerning the conventional compression method. The radiographers experienced the patient‐controlled method as less time‐consuming and more comfortable, but uncertainty about correct compression technique and its effect on radiation dose and image quality was reported.

## 1 INTRODUCTION

This study is part of a Swedish project evaluating a radiographer‐invented compression device. Compression is used for reducing radiation dose and improves image quality during conventional X‐ray examination. In the Nordic countries, the radiographer is a professional responsible both for the execution of the radiological examination—including keeping the radiation dose at a minimum and optimizing image quality—and for patient care throughout it.

## 2 BACKGROUND

All X‐ray examinations shall be justified, optimized and radiation doses set as low as reasonably achievable (ICRP, 2007), as radiation causes cancer and genetic damage (Radiation Protection Act, 2018). Reduction of radiation doses requires that the radiographer uses optimized methods for compression (Branderhorst et al., 2015). Research has shown that compression of a few centimetres can halve radiation dose. Compression is also used to improve image quality (Olsson, Tingberg, & Mattsson, 2010). There are traditionally two ways to compress the abdomen: one is that the radiographer adds a compression band across the abdomen and the other is that the patient is lying in a prone position. These compression methods can be used during all conventional X‐rays of the abdominal area. Prone compression is most frequently used, because it is simple. However, previous research has identified that it is not as effective as compression in the supine position (Piippo‐Huotari, Norrman, Anderzén‐Carlsson & Geijer, 2018; Olsson et al., 2010) and not all examinations can be done with the patient in the prone position. For thin patients, it has been shown that there is no compression at all in the prone position (Olsson et al., 2010). Despite benefits, compression can be experienced as painful and anxiety provoking and one study identified that reduced compression during digital breast tomosynthesis decreased both pain and anxiety, with maintained image quality (Abdullah Suhaimi, Mohamed, Ahmad, & Chelliah, 2015). Another, older study showed that patient‐controlled compression was less painful than technologist‐controlled compression and patient satisfaction was high and image quality was good (Kornguth et al., 1993).

Although the benefits of compression are known, in 2014 the Swedish Radiation Safety Authority identified low usage of compression in Swedish radiology departments—about 45% of all examinations (Larsson, 2015).

In the Nordic countries, the radiographer is a professional responsible both for conducting the radiological examination and caring for the patient during the procedure (Andersson, Fridlund, Elgan, & Axelsson, 2008). Radiographers have rated it as important to adapt the examination to the prerequisites and needs of the patient (Andersson, Christensson, Jakobsson, Fridlund, & Brostrom, 2012) and to protect the patient from unnecessary radiation (Andersson et al., 2008). Many studies show that communication between the radiographer and the patient can relieve patients' stress and anxiety associated with radiological examinations (Acuff, Bradley, Barlow, & Osborne, 2014; Andersson et al., 2008; Carlsson & Carlsson, 2013).

Because of the low use of compression in Swedish radiology departments and the clinical experience that compression is perceived as time‐consuming and complicated, and the knowledge that not all examinations can be done in the prone position, a patient‐controlled compression plate called the OIKE‐plate (design registration number: 82343) has been developed. It was tested in a previous study where we evaluated patient‐controlled compression compared with conventional compression. The study did not find any difference in radiation dose or image quality between the two methods (Piippo‐Huotari et al., 2018). It is, however, important to know the patients' and the radiographers' experiences of compression in general and of the new method specifically, to develop methods further and make compression devices as easy to use as possible. To the best of our knowledge, there is no previous study on experiences of compression from the radiographers' perspective or from a patient perspective, except for compression related to mammography.

The aim of the study was to describe the patients' and radiographers' experiences of compression of the abdomen using conventional compression methods and a patient‐controlled method.

## 3 METHODS

### 3.1 Design

The study has a descriptive design based on study‐specific questionnaires with patients having undergone intravenous urography including compression and interviews with the radiographers conducting these examinations. Data were analysed by qualitative content analysis.

### 3.2 Participants and setting

The study intervention took place at the radiology department at a university hospital in Sweden, from September 2015 to January 2016. In the intervention, three different compression techniques were performed during a standard clinical urography. These were as follows:

1. Conventional compression: The patient was positioned supine with the compression applied by the radiographer. A compression band was attached to both sides of the examination table and strapped over the abdomen with an extra pillow under the band.
2. Patient‐controlled compression: The OIKE‐plate compression device was used. The patient was told to hold and press the plastic compression plate against the abdomen after inhalation. An extra pillow was used between the abdomen and the device.
3. Prone position: The patient's body weight was used for compression.

The inclusion criteria for the patients in the main study were as follows: outpatients who were referred for urography and who were between 18–80 years old. The exclusion criteria were as follows: emergency patients, patients who could not participate actively, inability to understand the instructions or answer questions, contraindication for compression or pregnancy. Of the 50 patients who participated in the compression study (Piippo‐Huotari et al., 2018), 45 agreed to answer a questionnaire about compression in general, including specific questions about the conventional method, and the patient‐controlled compression. A total of 24 women and 21 men participated; their age was distributed as follows: age 18–33: *N* = 5, age 34–49: *N* = 10, age 50–65: *N* = 15, age 66–80: *N* = 15.

Information about the study, the invitation to participate and a consent form were sent together with the urography appointment. Written informed consent was obtained at the time of examination.

The other group of participants in this study are the radiographers who performed at least two examinations in the first study ( Piippo‐Huotari et al., 2018). The principal supervisor, after the completion of the first study, sent an e‐mail to all these radiographers with the invitation to participate in an interview study. Five agreed to participate. Three were women and two were men. They all had prior experience of the conventional compression method and had been responsible for 2–22 (median 5) of the examinations in the previous study, which is equivalent to 82% of all the examinations performed in the project. Written informed consent was obtained prior to the interviews.

### 3.3 Data collection

#### 3.3.1 Patient experiences

The patients answered the questionnaire at the radiology department after the urography examination. If patients were not able to answer the questions while they were there, they received a stamped envelope to be able to answer the questions at home and send the questionnaire to the radiology department.

The questionnaire was study‐specific and comprised two demographic questions, seven open‐ended questions concerning the experiences of the two compression methods, 10 questions where the alternatives were “yes,” “no” and “don't know” and a space for participants' comments. The latter questions were similarly phrased and concerned the two compression methods. They covered the areas of fear, pain, information needs, support from the radiographer and sense of security during the compression.

#### 3.3.2 Radiographers

Individual interviews were performed with the radiographers concerning their experience of compression related to X‐ray examinations in general and of the three different compression methods specifically. The interviews were performed by an experienced external radiographer (second author) to avoid undue influence of informants, because the first author has developed the patient‐controlled compression plate. The interviews were conducted from November 2016 to February 2017. They lasted between 17–31 min (median 22 min).

An interview guide was developed in the research group with the aim of covering the same topics studied in the patient survey, but from the radiographers' perspective. The interview was in the form of a conversation between the researcher and the radiographer, with individual follow‐up questions depending on the answers given, as recommended in the literature (Kvale & Brinkmann, 2009). They were conducted in a conference room at the radiology department, before or after the radiographers' ordinary work shifts. The interviews were audio‐recorded with the permission of the interviewee and later transcribed verbatim by an experienced secretary.

### 3.4 Data analysis

Both the qualitative part of the questionnaire and the interviews were analysed using qualitative content analysis according to steps described by Burnard (1991) and Burnard, Gill, Stewart, Treasure, and Chadwick (2008). The first author carried out analysis of the questionnaires in close cooperation with the last author. The second author did the analysis of the interviews in close cooperation with the last author. According to Burnard (1991), the close cooperation is important to ensure the validity of study results. Survey and interview data were analysed separately and are presented separately in Section 4.

#### 3.4.1 Patients' experience

Initially, all free text answers for each question in the survey were collapsed into one text (the unit of analysis). Thereafter, all text was read repeatedly to reach a more comprehensive understanding of the content. The text units corresponding to the purpose of the study were divided into different codes. In this step, text from various questions could be covered by one code, capturing a similar content. Codes with similar content were in the next step compiled into preliminary categories. Similar categories were then grouped together into broader categories, and finally, the text comprising the unit of analysis was re‐read and checked against the categorization to ensure that the categories were consistent with responses (Burnard, 1991).

#### 3.4.2 Radiographers' experience

The analysis of the interviews was conducted using inductive thematic content analysis following the steps described by Burnard et al. (2008). First, the interviewer listened to the interviews and at the same time also checked the accuracy of the transcripts and strived to get a deeper understanding of the content. In a second review of the interviews, the interviewer listened and took notes on content of importance for the aim of the study. In the next step, statements related to the aim were marked with various colour markers depending on the content and then sorted into preliminary categories. At this step, the text describing the advantages and disadvantages of the different compression methods was also extracted and inserted into a table to illustrate these results separately. Thereafter, a check of the preliminary categories was done with reference to the original transcripts. Finally, all text covered by a category was put together and after some reorganization to create a consistent structure in the categories, the results were written up.

Quotes from the interviews and questionnaires were used to illustrate the data and enhance the credibility of the findings (Graneheim & Lundman, 2004). The results are reported in line with the COREQ guidelines (Appendix S1).

### 3.5 Ethical consideration

The regional ethical review board in Uppsala has approved the study (registration number 2015/119 and 2015/119/1). The research followed the Declaration of Helsinki. The study participants received oral and written information about the study, the voluntary nature of participation and the right to withdrawal without having to specify the reason and without their decision having an impact on their examination (if they were patients). As the first author had invented the device used in the patient‐controlled compression, she was not directly involved in recruitment and implementation of surveys or interviews. The analysis of data was done in close collaboration by the first and the other authors to reduce the risk of bias.

## 4 RESULTS

First, the preferred method of compression from the perspective of the participating patients will be described. Thereafter, the patients' other experiences concerning compression are presented, and finally, the radiographers' perspective is focused on.

### 4.1 Patients' perspective

Most patients giving voice to which compression method they preferred stated that they preferred the patient‐controlled method (*N* = 18), while 12 preferred the conventional method. For another 10 patients, the method used did not matter. Five participants did not answer that question.

The patients who preferred the conventional compression stated that they believed that this method gave a more stable pressure, resulting in better image quality. They experienced it as more comfortable and easier and preferred the radiographer to be responsible for the compression. They felt uncertain about their own capacity to provide the optimal compression needed for quality images.

The patients who preferred the patient‐controlled method preferred to be in charge of the compression because of fear of pain due to excessive pressure. They could alter the pressure themselves to suit their own limits and this resulted in them not feeling restrained. Another aspect mentioned was that the total time with compression was shorter when using this method, as the pressure was eased immediately when the radiographer told the patient they could release it. It was more comfortable. One patient experienced it as easier to breathe using this method.

Of the 10 patients reporting that it made no difference to them which compression method was used, two elaborated on their standpoint, saying that they preferred the method that ended in the best quality images (Table 1).

**Table 1:** The distribution of answers given for the closed questions in the questionnaire

	*N*	%
Having been informed of reason for compression	34 yes, 6 no, 5 do not know, 0 no answer	76/13/11/0

	Conventional compression		Patient‐controlled compression	
	*N*	%	*N*	%
Fear of compression	1 yes, 44 no	2/98/0/0	45 no	0/100/0/0
Pain related to compression	4 yes, 40 no, 1 does not know	9/89/2/0	3 yes, 41 no, 1 does not know	7/91/2/0
Adequate support when undergoing compression	43 yes, 0 no, 1 does not know, 1 no answer	96/0/2/2	44 yes, 0 no, 1 does not know	98/0/2/0
Feeling safe during compression	43 yes, 0 no, 1 does not know, 1 no answer	96/0/2/2	43 yes, 1 no, 1 does not know	96/2/2/0

The findings from the content analysis of the compression during the urography are presented below under six themes: Unproblematic and quick; Being well‐informed and taken care of; Being involved and in control; Patient safety; Feeling discomfort; and Radiographer‐focused concerns.

#### 4.1.1 Unproblematic and quick

Most patients reported the compression during the examination as unproblematic, both in general and when using either method for compression. Some described that this phase of the examination went quickly and that this correlated with the experience of the compression as being unproblematic: “Since the pressure on the abdomen lasted so short a time, there was no problem coping with it” (P29). It was described as easy to undergo compression, as it was just a matter of following the instructions given. Some of the patients mentioned that the compression did not cause them too much discomfort. The reasons for favouring one or the other method were somewhat similar.

#### 4.1.2 Being well‐informed and taken care of

The patients described themselves as being well‐informed about the examination and the compression methods used in the examination.

They appreciated the verbal and the written information that they had been offered and that the radiographer explained the procedure in a pedagogic and detailed manner. Information was given about how the procedure was to be performed and what was expected of the patient: “The staff were thorough when informing me about the procedure and I felt secure in their hands” (P24). Some radiographers showed the equipment when they explained the procedure and some kept patients continually informed during the entire examination. The information given was almost always easy to comprehend, and the patients said that the radiographers were careful that the patient had understood the information, which in turn made the patient feel secure. Some patients described asking questions in addition to the information given.

It was regarded as important, prior to the examination, to have information about the implications of the need for compression during urography. The patients described that they needed information about what to do during the examination, about the right to decide on how much compression should be applied, about the duration of the compression, advice about appropriate breathing technique during compression and that the bladder should be emptied prior to the examination. Some specifically mentioned that it was important to have information sent home in advance, to better prepare for the examination and in case patients had abdominal problems or a phobia of being restrained.

With regard to the conventional compression method, the patients specifically wanted honest information about the fact that the compression involved a hard pressure on the abdomen, but only for a short time: “How long you were supposed to be ‘stuck’ and that there will be severe pressure for a short while” (P35). It was also regarded as important that the patient was informed that they could tell the radiographer in the event the pressure was experienced as too hard and that the radiographer could rapidly loosen the compression in cases where the patient felt restrained. With regard to the patient‐controlled compression, the only specific need for information was to be told what to do when responsible for the compression, and feedback about how well they had achieved the compression.

The patients described the radiographers' encounters with them as professional: “Incredibly professional care by a fantastic radiographer” (P7). They appreciated when the radiographer showed them respect and was responsive. The patients felt safe when the radiographer acted in a professional and knowledgeable manner, indicating they knew what they were doing. They also appreciated when the radiographer acted in a calm way and informed the patients and answered their questions.

#### 4.1.3 Being involved and in control

The patients described feeling involved and in control of their care with regard to both compression methods.

Receiving adequate information was central for feeling involved. The patients said that they could endure the examination better if they were in control and by deciding the degree of compression: “It was all right when I could decide myself when the compression became unbearable” (P7). Similarly, one patient explained: “It increased in pressure step by step according to what I felt I could handle” (P29). It was described as easier to endure the compression if the breathing technique—using shallower breathing and not including the abdominal muscles—was adopted. What was experienced as threatening the sense of control was the inability to decide on the degree of pressure during compression, not being able to anticipate how hard the pressure would be and feeling restrained, and non‐awareness of the duration of the pressure, during compression with the conventional method.

The patient‐controlled mode of compression was frequently described in terms of the patient having control and being involved in care, as the patients themselves held the compression device and decided on the degree of pressure. It was easier to relax and endure the examination when controlling the pressure and the patients felt less restrained. The pressure was itself experienced as more comfortable and less demanding when the patients felt they were in control. Some found that they applied even more compression when they controlled the device compared with the conventional method. It was also experienced as a good feeling to be responsible for one specific task during the examination. It made the patient feel more involved: “Good to be able to participate in the examination, so simple” (P45).

#### 4.1.4 Patient safety

This category focuses on patient safety, as experienced by the patients. Mostly, this was related to their own ability to compress the abdomen, but there were also some statements about how the radiographers' behaviour made them feel safe.

The patients described feeling safe when using conventional compression, as the pressure was harder, more stable and evenly distributed, which they guessed could be better for the image quality: “The pressure on the abdomen gets better. A feeling that the results [of the images] will be better” (P20).

The patient‐controlled compression was more comfortable, but the participants were uncertain whether they could create the even pressure that was needed, how much pressure was needed and whether they handled the device correctly. They were worried that the images would not be of sufficient quality: “It felt good. I pressed as much as I could, but I was a bit unsure whether it was enough for a good result” (P24). Another patient explained:

I could summon more force in my arms and press the plate downwards harder than I thought beforehand. [Initially] the feeling was that I couldn't press as hard as with the other type of compression that was attached to the examination table. (P29)

The participants identified that it could be potentially hard for weak patients to press the device hard enough and hard to both press the device towards the abdomen and breathe at the same time. One patient worried about the image quality, as their hands were shaking when using the patient‐controlled device. One patient found that the device was too large for her abdomen, which created some insecurity about the quality of the examination. On the other hand, some believed that they could press even harder than the conventional compression method. One patient believed that the pressure was less evenly distributed with the conventional device, as it was looser at the side where the device was fastened.

#### 4.1.5 Feeling discomfort

The discomfort experienced was often described in terms of pain and unpleasantness of the compression of the abdomen. The discomfort was more often related to the conventional compression method. The discomfort ranged from a slight unpleasantness to sheer pain.

The main discomfort described with the conventional compression method was pain and experiencing that it was hard to breathe. As described under the heading *Being involved and in control*, the discomfort was also related to enduring the feeling of being restrained. One patient stated: “When you don't control it yourself it is frightening. Thoughts like ‘what if it is pressed really hard’ – this might be a torture method, with a little more pressure” (P35). Only one participant described discomfort in relation to the patient‐controlled compression.

#### 4.1.6 Radiographer‐focused concerns

Some patients also commented on the situation of the radiographers and the organization. This was exemplified in terms of the conventional compression method as inconvenient for the radiographer to carry out. The patient‐controlled method was described as easier for all parties. It seemed to be more effective, which is good for the patient and the healthcare organization. One patient had been given the impression that the patient‐controlled method implied less radiation doses for the radiographer, which was regarded as a positive aspect.

### 4.2 Radiographers' perspective

The radiographers were well aware that the reason for using compression was to decrease the radiation dose to the patient and to enhance the image quality. They were also aware of their responsibility to use it. “I know that it reduces the radiation dose and we also get better images” (R1). Although the radiographers described the necessity of it, compression in general (conventional technique) was experienced as an extra time‐consuming task that was tedious and tiresome “An extra procedure that must be done and it is cumbersome” (R4).

The results from the thematic content analysis are described below under the headings *Working environment*, *Caring for the patient during the examination*, *Patient safety* and *Documentation about abdominal compression*. The advantages and disadvantages experienced with the conventional compression method and the patient‐controlled method are summarized in Table 2.

**Table 2:** Identified advantages and disadvantages of the two compression techniques, from the radiographers' perspective

Conventional compression		Patient‐controlled compression	
Advantages	Disadvantages	Advantages	Disadvantages
Better control for the radiographer (safer, more reliable)			Less control for the radiographer
		More control/ participation for the patient	
	Time‐consuming (forms queues)	Takes less time (less stress)	
	Awkward and tiresome	Easy to use	
	Easy to “forget”	More radiographers would probably use it	
Higher compression pressure?			Lower compression pressure?
		Opportunity to compress on more images	

#### 4.2.1 Working environment

The workload in the radiographic department was experienced as having an impact on whether the radiographers felt they had time to use compression, even though this procedure was in accordance with the local guidelines. Another aspect mentioned was the ergonomic strain for the radiographers. They often felt pressured to conduct the examinations quickly, to keep the waiting time for patients short. This was especially mentioned in relation to examinations in the drop‐in laboratory:

There is often a pressure to perform the examinations as quickly as possible, so if you have a fixed schedule and if you have time there is no problem performing all steps of compression, but if there are drop‐in examinations, they should simply be performed as rapidly as possible, to avoid too‐long waiting lines. (R3)

Due to time restraints, this meant that they sometimes did not use compression during the examinations: “You don't have the time to attach the device, things should happen as fast as possible” (R4). A similar point, related to time pressures, was that the radiographers wanted the compression to be easy to perform. Sometimes, the radiographers described forgetting compression due to a heavy workload, although they tried to comply with the routine: “Sometimes you forget to use the compression devices when there is too much to do, but at least you try to remember it” (R4).

Concerning the two techniques, the radiographers describe the conventional technique as time‐consuming, with many steps, like retrieving and attaching the compression equipment at the back of the table, walking around to the other side and tightening the device. Two radiographers suggested having the conventional compression device permanently attached to the examination table, as this would make the equipment easier to use. “The device that you attach to the side would be good to have attached all the time, to have it on the table, just to pull out” (R2).

The conventional compression technique was also described as a less ergonomic way to work, as the radiographers have to lean over the wide examination table to reach the compression belt, especially when examining large patients:

If the patient is large, as with the conventional compression, it could be, for me ergonomically it is not very good having to reach over the patient to try and reach this [the compression device] on the other side, we have quite wide tables, so it could be technically difficult. (R1)

Thus, the patient‐controlled compression was experienced as being quicker and easier to use: “It is much easier to use for us radiographers. It hardly takes any extra time, just give them the plate and let them pull” (R5).

#### 4.2.2 Caring for the patient during the examination

To observe and communicate with the patient during the entire examination and while compressing was described as essential, regardless of technique:

And then I advance the tension a few clicks, it is like different levels. And then you have to check the patient, to see how they react. If you see that they are affected, then perhaps you don't press as hard. Some people tolerate quite a lot, so then I can pull hard. (R4)

Likewise, if the patient was in pain, the radiographer might decide to omit compression. In a similar vein, it was suggested that if the patient had problems with compression, they could compress themselves by pulling in the stomach without any compression at all:

Instead of having nothing at all, if you tell the patient to pull in the stomach as much as they can, you can be quite thin just by pulling in, without even compressing. (R3)

Some of the radiographers were under the impression that the patients, who were part of the randomized trial and thus using both compression techniques, preferred the patient‐controlled compression. They listed two major reasons for this: first, it meant the patient was compressed for a shorter time, because the radiographer can tell the patient to release the pressure immediately after the image is taken, compared with the conventional technique where the radiographer has to enter the room after the image is taken and release the compression manually. Second, the patients can decide themselves how hard to compress. “The most comfortable [for the patients] is definitely the patient‐controlled device, because it doesn't press so hard” (R3).

The radiographers believed that giving good information to the patient about the examination is the alpha and omega; what is going to happen, how and why:

I think that when you explain that it is used to reduce the radiation sort of, I believe they feel it in a positive way; some consider it troublesome when it is tensioned; still the ones I have encountered have experienced it positively. (R4)

Some of the radiographers also said that they, in connection with the oral information, also showed the procedure visually to the patient: “then I show” (R2). One radiographer tested the compression practically together with the patient prior to the examination procedure taking place: “I usually instruct and try it once” (R1). They also described that the information given to patients can vary from time to time and from patient to patient and that it is important to get a feeling for the patient to know when to do something.

The radiographers experienced that the patient‐controlled compression implied a need for more detailed information because it requires the patient taking a more active role. It is therefore necessary that the patients can understand and follow instructions. “The patient must understand what to do and be able to cooperate” (R1).

Regardless of compression technique, the experience was that some patients tended to push out the abdomen when inhaling. Thus, they might need extra information explaining that they should make themselves as flat as possible during the compression; otherwise, the radiation dose reduction and the image quality would not be optimal. “‘You need to be as flat as possible, then it is good if you pull in your abdomen’. Some almost push their abdomen outwards when inhaling” (R3).

#### 4.2.3 Patient safety

Although the patient‐controlled technique was regarded as preferable in terms of workload and ergonomics, the radiographers were aware that they had to take the patient's ability to compress into consideration when choosing which compression technique to use: “I would use the patient‐controlled device if I see that the patient can handle it, because it is easier to handle!” (R1).

Nevertheless, some radiographers preferred the conventional technique because it felt more reliable and safer to use, as they then had better control: “Because then I can press hard, then I can decide the pressure” (R2) and “I believe I think that the conventional compression where you pull the band across the abdomen that I adjust myself works a little better, because I know that some don't really understand how much they have to press” (R4).

Although the conventional technique was regarded as more reliable due to a professional compression, the radiographers believed that the degree of compression varied from radiographer to radiographer:

It feels quite individual, from radiographer to radiographer, if I control the compression I might compress more or less than my colleague, since we have different opinions on what is suitable. (R3)

Similarly, the patient‐controlled compression could vary depending on how hard the patient compressed themselves, which could have an impact on the level of compression achieved: “But you don't know how much [compression] the patient uses, so then you have to trust that the patient, well, understands and does what he is able to” (R2) and “It is hard to tell, but the feeling is that there is slightly less pressure when they compress themselves, I think” (R5).

The patient‐controlled technique resulted in the radiographers suggesting further improvements to the new device, as they had identified some shortcomings. It may be difficult for slim patients to compress themselves sufficiently, as their elbows then will touch the examination table unless they angle their elbows, which would result in low compression. Likewise, the breadth of the examination table could be an obstacle for self‐compression. One solution given was to put a thick pillow on the patient's stomach: “When they were too thin, they had problems – perhaps you could have a higher pillow – when they were supine and should compress, the elbows sort of get stuck” (R1). One radiographer had observed that for some patients, the handles on the plate were too close to each other, presenting the risk that they could be visible on the images and therefore suggested more than one size of compression plate. “The plastic handles are placed too close together, so they might be visible in the images; there should be a few different sizes” (R5).

One bonus with the patient‐controlled technique that was described was that it is possible to use compression for more images—not just frontal images, but also for the oblique projections. “When I use the patient‐controlled device I can use compression also in the oblique projections” (R1).

One radiographer believed that even if the radiation dose were to be slightly higher with the new technique, it is likely that more radiographers would actually use that technique compared with the conventional technique, so to summarize, it would benefit the patient:

We have such a high patient flow, that I think it is a good complement [the patient‐controlled device] to get all us radiographers to actually use compression in a simple way. (R1)

#### 4.2.4 Documentation about abdominal compression

Although the radiographers were aware of their responsibility to document when compression had been used during examinations, they admitted that they sometimes forgot to tick the box that the Swedish Radiation Safety Authority use for national statistics. As a consequence, the current statistics were regarded as unreliable:

I try to enter the information that the Radiation Safety Authority needs, that we actually [use compression]; it is easy to forget, so when you are conscientious and use compression, you forget to fill in this information. (R1)

If the patient cannot be compressed, the reason for this should be noted: “then I enter ‘no, I couldn't apply compression’ and then I have to enter why; I just write something short, like pain or prosthesis or fracture” (R1).

## 5 DISCUSSION

The patients to some degree offered similar explanations for their preference of one or the other method of compression. However, it was only with regard to the patient‐controlled technique that they mentioned benefits for the radiographers. They regarded this method as more ergonomic and that by using it the examination went more quickly. These aspects were also mentioned by the radiographers. The Swedish Radiation Safety Authority has identified that compression is used to a lesser degree than prescribed (Larsson, 2015). However, the basis for their assumption could be wrong, as the results in this study indicate that the radiographers do not always remember to document that they have used compression. On the other hand, the results also indicate that the radiographers are of the opinion that they do not always prioritize compression when working under intense pressure. Perhaps introducing a simple patient‐controlled method for compression, as described in this study, could result in better adherence to the guidelines related to using compression in radiology, as it was experienced as more convenient to use. However, further studies are needed to measure examination time, cost benefits and study ergonomics specifically when using the new method.

Both the patients and the radiographers believed that the conventional compression method was safer and resulted in a decreased radiation dose and increased image quality compared with the patient‐controlled compression. Swedish legislation prescribes that the radiation dose should be kept to an absolute minimum (Radiation Protection Act, 2018), and a previous study concluded that the two compression methods described here are similar with regard to radiation dose and image quality (Piippo‐Huotari et al., 2018). Balleyguier's study showed that compression was better with patient‐assisted compression, and image quality was equivalent compared with technologist‐assisted compression (Balleyguier et al., 2018). It is, however, important to offer education to the radiographers when introducing a new method, to assure an optimal use of the equipment and techniques and to maintain low radiation doses. It is also important to inform patients thoroughly about how to compress their abdomen to achieve an optimal compression and for them to feel confident about their own contribution. According to previous studies, information can help patients understand the procedure (Hellman & Lindgren, 2014) and the interaction with radiographers can help patients endure examinations (Carlsson & Carlsson, 2013).

In this study, as in previous literature (Ukkola et al., 2016) patients have asked for information about the risks related to radiation when undergoing X‐ray examinations. Furthermore, patients have expressed a wish to actively participate and make informed decisions when there are alternatives (Ukkola et al., 2016). To choose between two compression methods could be a decision that the patient can make, as the two methods described here have been shown to be comparable with regard to image quality and radiation dose. To increase a sense of participation further, perhaps it would be a good idea to send information by mail prior to the examination, for example when sending the appointment. This is in line with the conclusions made by Carlsson and Carlsson (2013) with regard to MRI.

Some of the patients experienced that the compression was painful, had feelings of being trapped and suffered anxiety. This was most often mentioned in relation to the conventional compression. The radiographers were aware that compression could cause pain and thus they were observant of this when using compression and some suggested that the patient‐controlled compression could enable a smoother procedure in such cases. A previous study from a mammography setting showed that a reduced compression force reduced anxiety and pain levels without compromising image quality (Abdullah Suhaimi et al., 2015). Balleyguier et al. showed that patient‐assisted compression induced significantly higher compression, but it did not increase discomfort or pain (Balleyguier et al., 2018). Thus, it is likely that the patient‐controlled compression method could be an alternative for patients in pain.

The patient‐controlled compression could also increase participation in the examination, as the patient can decide on the level of pressure and is offered a task to perform. The value of participation has previously been mentioned, related to the patient‐initiated breath‐holding technique during MRI (Funk, Thunberg, & Anderzen‐Carlsson, 2014). Adequate information that allows the patient to get involved in their own examination has been shown to give the patient a sense of control and could result in a satisfactory examination (Andersson et al., 2008; Carlsson & Carlsson, 2013). According to Balleyguier et al. (2018), the patients said that they had control over the procedure, and they could manage their own stress and take part in examination with the patient‐assisted compression. Previously, Andersson et al. (2008) found that radiographers rated themselves as not very good at including patients in quality improvement. This study is, however, an example of how to involve patients in such work. Kornguth describes that with minimal patient education, self‐compression could provide images as good as those from technologist‐applied compression (Kornguth et al., 1993).

The results show that some patients found it difficult to maintain the compression due to muscle weakness in their arms. In line with this, the radiographers suggested the new device be further developed with regard to the handles. It is important to evaluate new devices before taking them into ordinary use. This can be done by measuring effect and by asking the users for their opinions (Goodman, 2014), as in this study.

### 5.1 Strengths and limitations

The novelty of the topic of this study, describing patients' and radiographers' experience of compression, is in itself one major strength. Another strength is the data triangulation methodology (Polit & Beck, 2012), where the experiences from both patients and radiographers were collected, which in many cases supported and complemented each other.

It is a strength that almost all invited patients decided to participate in the questionnaire part of this study. Most of the patients wrote only one line or less, but because 45 of 50 invited patients enrolled in the study, the data were regarded as rich enough to answer the purpose. With regard to the radiographers' perspective, it would have been valuable to include some more radiographers with previous experience of examinations including compression, as this could have resulted in additional perspectives. However, we decided to restrict the sampling to those who had been involved in the study‐specific examinations, because of the purpose of the study.

## 6 CONCLUSION AND RELEVANCE TO CLINICAL PRACTICE

This study adds to the knowledge on experiences related to compression. The fear of pain and being strapped down were central for the patients, as was the image quality. For the radiographers, the new method was more ergonomic and made the examinations quicker, but the new method also made them uncertain of the image quality and radiation dose, as they lacked control over the compression. With the knowledge that the results of the two methods are comparable, perhaps the new method could lead to more radiographers actually using compression, in accordance with guidelines, as it was experienced as more convenient. This in turn would benefit the patients, as it will decrease the radiation dose and increase image quality. Perhaps the new method can be used in imaging where it is not possible to use the conventional method, such as imaging in a standing position.

## CONFLICTS OF INTEREST

The first author has developed the OIKE‐plate. Thus, she was not involved in the data collection. She participated in the data analysis, under supervision of the last author. The authors deny any other conflicts of interest.

## AUTHOR CONTRIBUTIONS

OP‐H, HG and AA‐C: designed the study. EF: conducted the interviews. OP‐H, EF and AA‐C: conducted the analysis. OP‐H, EF, HG and AA‐C: drafted the manuscript and agreed on the final version.
